# Regional, demographic, and temporal trends in psychoactive substance use-related mental disorder and cancer mortality in U.S. adults: a nationwide CDC WONDER analysis (1999–2020)

**DOI:** 10.3389/fonc.2026.1753582

**Published:** 2026-03-18

**Authors:** Asma Chaudhary, Muhammad Sarim Azad Khan, Ibrahiem Azeem Ajaz, Aroosha Waheed, Arham Khalid, Mirha Imran Khan, Aizaz Anwar Khalid, Aisha Chaudhary, Saqib Raza Khan, Mohammed Hammad Jaber Amin

**Affiliations:** 1 Department of Medicine, Fazaia Medical College, Islamabad, Pakistan; 2 Department of Medicine, Combined Military Hospital Lahore Medical College, Lahore, Pakistan; 3 Department of Medicine, Rashid Latif Medical College, Lahore, Pakistan; 4 Department of Medicine, Rawalpindi Medical University, Rawalpindi, Pakistan; 5 Department of Medicine, Combined Military Hospital Institute of Medical Science, Multan, Pakistan; 6 Department of Medicine, Peshawar Medical College, Peshawar, Pakistan; 7 Verspeeten Family Cancer Centre, London Health Sciences Centre, London, ON, Canada; 8 Department of Oncology, Division of Medical Oncology, Schulich School of Medicine and Dentistry, Western University, London, ON, Canada; 9 Department of Medicine, Alzaiem Alazhari University, Khartoum, Sudan

**Keywords:** age-adjusted mortality rate, cancer, mortality trends, psychoactive substance-related disorders, substance use

## Abstract

**Background:**

Cancer remains a major global health burden, with 10 million deaths in 2020 and among the 35 million adults worldwide with psychoactive substance use disorders (SUDs), it is a major contributor to premature mortality. In the U.S., tobacco causes over 30% of cancer deaths and alcohol nearly 5%, highlighting the role of modifiable behaviors. Despite this clinical and economic burden, national long-term data on co-occurring SUDs and cancer mortality remain limited. We therefore analyzed 22 years (1999–2020) of U.S. mortality data from CDC WONDER (Centers for Disease Control and Prevention Wide-Ranging Online Data for Epidemiologic Research) to evaluate temporal trends and demographic and geographic disparities in psychoactive substance–related mental disorder mortality in relation to cancer among adults aged ≥25 years.

**Methods:**

Mortality data for adults aged ≥25 were obtained from CDC WONDER using ICD-10 codes C00–D48 (cancer) and F10–F19 (psychoactive substance–related mental disorders). We calculated age-adjusted mortality rates (AAMRs) and annual percent changes (APCs) by demographics and region. Subgroup analyses paired cancer deaths with alcohol, tobacco, and other psychoactive substance categories to assess substance-specific trends.

**Results:**

From 1999–2020, 1,789,591 deaths involved both cancer and substance use (SU)–related mental disorders. Overall AAMR rose from 5.66 in 1999 to 43.26 in 2020, increasing sharply from 1999–2005 (APC: 41.67), more gradually through 2012 (APC: 4.36), and stabilizing thereafter (APC: –1.23; p>0.05). Rates were higher in males than females (51.57 *vs*. 25.8), highest in non-Hispanic (NH) American Indian/Alaska Native individuals, and lowest in NH Asian/Pacific Islanders. Regionally, AAMR was greatest in the Midwest (49.32) and lowest in the West (25.38) with Vermont and California representing the highest and lowest AAMR states, respectively. Nonmetropolitan areas had higher rates than metropolitan areas (49.6 *vs*. 34.26). By substance, alcohol-related AAMR declined until 2012 then rose, tobacco increased sharply early and later stabilized, and “psychoactive drug”–related mortality was stable until 2008 before increasing.

**Conclusion:**

Overall, AAMR plateaued after 2012, with the highest burden in NH American Indian/Alaska Natives, males, and rural Midwest residents, highlighting demographic and geographic disparities among individuals with concomitant cancer and psychoactive substance use–related mental disorders.

## Highlights

- Psychoactive substance use -related cancer mortality rose from 1999 to 2012, then plateaued through 2020.
- Men consistently had higher mortality than women.
- Highest AAMR was observed in NH American Indian/Alaska Native & NH White; lowest in NH Asian/Pacific Islander.
- The Midwest and certain states (VT,MT,NE) had the highest mortality burdens.
- Among psychoactive substances, tobacco was linked to the highest AAMR in association with cancer.

## 1 Introduction

Cancer is a leading cause of morbidity and mortality worldwide, with 19.3 million new cases and 10 million deaths in 2020 (1). Globally, 35.6 million adults live with psychoactive substance use disorders (SUDs), among whom cancer contributes substantially to life-years lost, up to 24 years (2, 3). In the United States, smoking accounts for over 30% of cancer deaths, and alcohol is associated with 4.8% of cases (2013–2016) (4, 5), highlighting the impact of modifiable behaviors. Many individuals with SUDs have chronic comorbidities, severe mental illness, and engage in high-risk behaviors such as smoking, poor diet, physical inactivity, and low participation in cancer screening (6), contributing to disproportionately high cancer mortality, particularly among males and Non-Hispanic (NH) American Indian populations (7). Psychoactive substances increase cancer risk and mortality through carcinogenic, biological, and behavioral pathways, while neuroadaptive changes associated with addiction exacerbate mental illness and accidental death. Among these substances, alcohol and tobacco, both classified as Group 1 carcinogens, remain major drivers of preventable cancer deaths (8). Although tobacco use and related mortality declined during the 20th century (9), tobacco-related cancers still affect millions (5), and broader SU, including alcohol, opioids, and other psychoactive drugs continues to drive substantial health disparities despite global initiatives such as the UN Sustainable Development Goals (10, 11). These substances contribute to higher cancer incidence and reduce survival (12, 13), posing an ongoing public health challenge.

Between 2015 and 2020, U.S. cancer care costs grew by about 10%, largely due to population growth and aging (14). Substance use (SU), particularly tobacco, drives significant cancer-related mortality, resulting in millions of lost life-years and billions in lost earnings, further increasing the economic burden of cancer (5). While prior research has described SUDs and mental health disorders in cancer populations and vice versa, these studies are often limited to selected physical conditions, institutional cohorts, registries, or survey-based data, which do not capture the full U.S. population (6, 12) and may underrepresent rural or underserved areas, potentially underestimating geographic and demographic disparities. Comprehensive, longitudinal mortality data linking cancer and SUDs remain scarce, and most existing studies provide only cross-sectional or partial insights into co-occurring conditions (15–17). To address this gap, we conducted a 22-year (1999–2020) population-based analysis using CDC WONDER (Centers for Disease Control and Prevention Wide-Ranging Online Data for Epidemiologic Research), which captures virtually all deaths in the United States. By examining age-adjusted mortality rates (AAMRs) stratified by sex, race/ethnicity, geographic region, and substance type, this study provides the first nationwide assessment of temporal trends, demographic and geographic disparities, and the distribution of psychoactive substance–related mental disorder mortality in association to cancer. These findings offer descriptive insights to guide prevention and survivorship care for populations at highest risk.

## 2 Methodology

### 2.1 Study setting and population

This study utilized mortality data from the CDC WONDER database, a widely used and authoritative source for analyzing AAMRs across the U.S. population (18). The platform allows detailed stratification by demographic and geographic variables, including sex, race/ethnicity, states, 2013 urbanization classification, and census region, facilitating the identification of high-risk subpopulations. We used the “Multiple Cause of Death Public Use” dataset, which captures all deaths in which cancer or psychoactive SU–related mental disorders were listed as either the underlying cause or a contributing cause of death.

Mortality data were extracted for 1999–2020 using 10th Revision of the International Classification of Diseases (ICD-10) codes; C00–D48 for cancer and F10–F19 for psychoactive SU–related mental disorders. For subgroup analyses, cancer-related deaths (C00–D48) were paired separately with substance category to assess substance-specific mortality trends: alcohol (F10), tobacco (F17), and other psychoactive substances (F19). This dataset, which has been extensively used in related epidemiologic research (19, 20), includes death certificate records from all 50 U.S. states and the District of Columbia and focuses on adults aged ≥25 years. Because all data were publicly available and fully de-identified, Institutional Review Board (IRB) approval was not required. The study followed the Strengthening the Reporting of Observational Studies in Epidemiology (STROBE) guidelines to ensure methodological transparency and reporting rigor (21).

### 2.2 Data extraction

The data were obtained for this study on various mortality-related variables that included population size, year, place of death, urban-rural stratification, regional delineation, and specific classification to each state. Demographic variables included sex, race/ethnicity. Race/ethnicity was classified as NH White, NH Black or African American, Hispanic or Latino, NH American Indian or Alaskan Native, and NH Asian or Pacific Islander. Place of death was categorized as occurring in medical facilities (outpatient, emergency room, inpatient, death on arrival, or unknown), at home, in hospice, or nursing home/long-term care settings. Urban–rural classification followed the 2013 National Center for Health Statistics scheme, defining counties as urban (large metro ≥1 million; medium/small metro 50,000–999,999) or rural (<50,000) (22). Geographic regions were assigned per U.S. Census Bureau definitions: Northeast, Midwest, South, and West (23).

### 2.3 Statistical analysis

To evaluate national trends in cancer and psychoactive SU–related mental disorder mortality, we calculated crude mortality rates (CMRs) and AAMRs with corresponding 95% confidence intervals (CIs) per 100,000 population from 1999 to 2020. CMRs, presented in the **Supplementary File**, were derived by dividing the annual number of deaths attributed to cancer and psychoactive SU–related mental disorders by the respective U.S. population for each year. AAMRs were standardized to the 2000 U.S. standard population, and all main-text analyses were based on AAMRs to ensure consistency and comparability across demographic groups and over time. Temporal trends were assessed using the Joinpoint Regression Program (Version 5.4.0, National Cancer Institute) (24). Log-linear regression models were fitted beginning with the simplest model, adding joinpoints iteratively based on model fit criteria. The annual percentage change (APC) and its 95% CI were estimated for each identified trend segment. APC values were expressed as positive or negative based on the direction of the slope, indicating increasing or decreasing mortality trends, respectively. Statistical significance of APC estimates was evaluated using a two-tailed t-test, with p < 0.05 considered significant. All mortality counts, AAMRs, standard errors, and annual population estimates for 1999–2020 were obtained from the CDC WONDER database.

## 3 Results

A total of 1,789,591 deaths attributed to cancer and psychoactive SU-related mental disorder in the United States between 1999 and 2020 (**Table 1****).** Most deaths were recorded at home (42.17%), followed by medical facilities (31.21%), nursing homes (11.66%), and hospice settings (10.27%)**. (****Supplementary Table 1**).

**Table 1:** Cancer and psychoactive substance use–related mental disorders deaths, stratified by sex and race in the United States, 1999 to 2020.

Year	Overall	Women	Men	Hispanic/Latino	NH American Indian or Alaska Native	NH Asian or Pacific Islander	NH Black or African American	NH White	Population
1999	9995	3657	6338	331	78	58	1189	8310	180408769
2000	13851	5060	8791	368	95	87	1445	11804	181984640
2001	13458	5025	8433	398	120	85	1282	11536	184305128
2002	13693	5158	8535	390	85	97	1306	11772	186208028
2003	37399	14304	23095	1338	213	241	3462	32073	188090429
2004	52532	20289	32243	1573	416	359	4571	45528	190205384
2005	67745	26000	41745	1990	427	388	5991	58861	192551384
2006	73597	28511	45086	2083	443	708	6332	63878	195019359
2007	80611	31568	49043	2082	500	781	7030	70114	197403777
2008	89741	35067	54674	2320	512	854	7939	77935	199795090
2009	89250	34792	54458	2264	526	830	7768	77666	202107016
2010	98053	37973	60080	2508	617	1014	8769	84911	203891983
2011	102043	39551	62492	2593	607	986	8967	88712	206592936
2012	109644	42367	67277	2862	621	1039	9655	95185	208826037
2013	110301	42527	67774	3018	596	1079	9925	95454	211085314
2014	112646	43298	69348	3123	740	1086	10567	96860	213809280
2015	116694	45215	71479	3168	747	1210	10783	100382	216553817
2016	118261	45761	72500	3432	834	1335	11118	101215	218641417
2017	119140	46171	72969	3511	829	1292	11223	101967	221447331
2018	120254	46586	73668	3637	787	1258	11240	103049	223311190
2019	120831	46619	74212	3903	850	1429	11593	102813	224981167
2020	119852	46275	73577	3815	834	1459	11367	102142	226635013
Total	1789591	691774	1097817	50707	11477	17675	163522	1542167	4473854489

### 3.1 Annual trends in AAMRs for psychoactive substance use-related mental disorders and cancer

**Figure 1:**
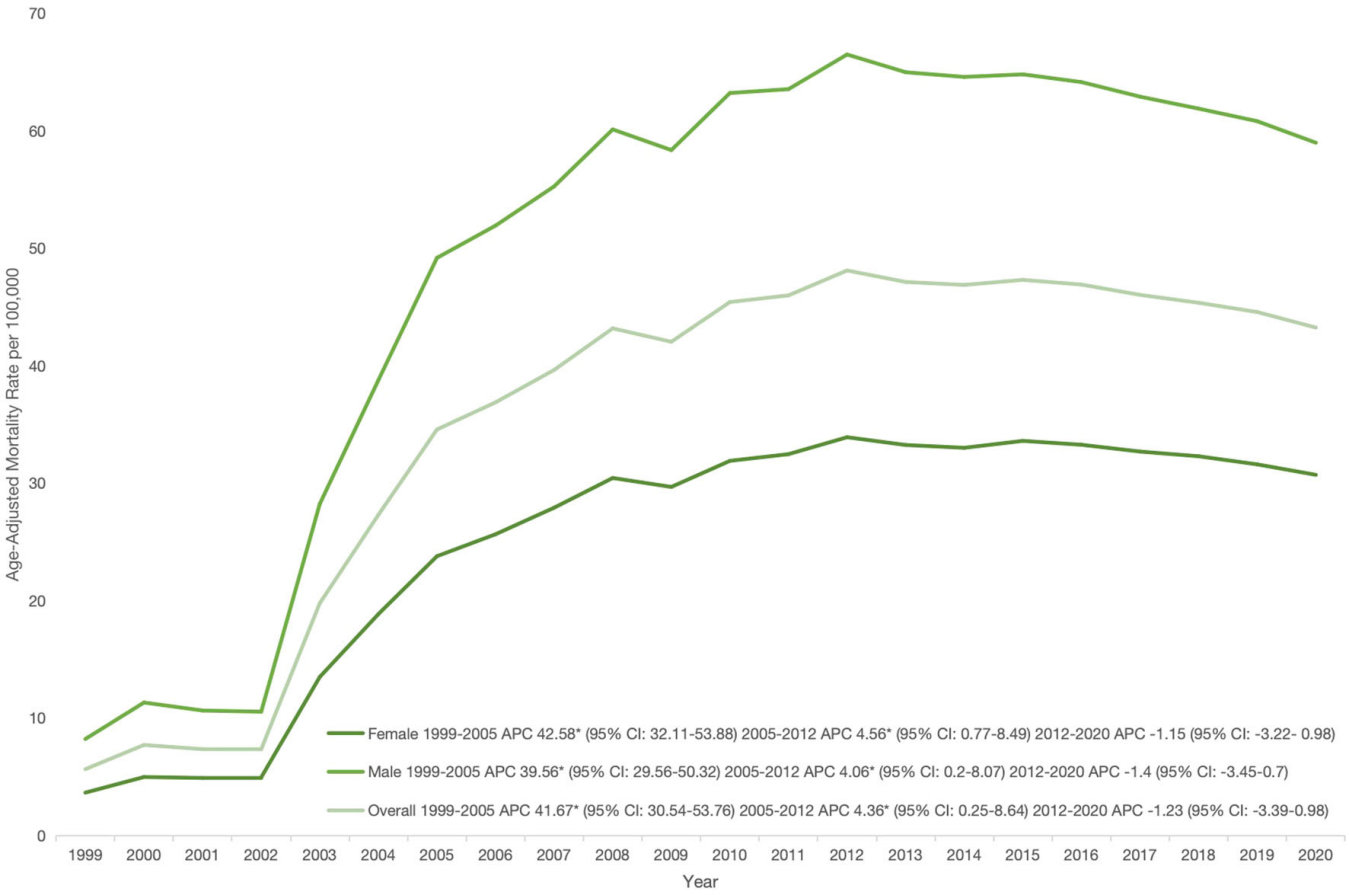
Overall and sex-stratified age-adjusted mortality rates per 100,000 for psychoactive substance use–related mental disorders and cancer among U.S. adults aged ≥25 years, 1999–2020. *Indicates that the annual percentage change (APC) is significantly different from zero at α, 0.05; APC, annual percent change; CI, confidence interval..

Overall AAMR ranged from 5.66 (95% CI: 5.54–5.77) in 1999 to a peak of 43.26 (95% CI: 43.01-43.51) in 2020. AAMR rose sharply from 1999 to 2005 (APC: 41.67; 95% CI: 30.54–53.76), increased more gradually until 2012 (APC: 4.36; 95% CI: 0.25–8.64), and then stabilized through 2020 (APC: –1.23; 95% CI: –3.39–0.98; p>0.05). (**Figure 1**; **Tables 2**, **3****).**

**Table 2:** Annual percent change (APC) for cancer and psychoactive substance use related age-adjusted mortality rates (AAMR) per 100,000 in the United States, 1999 to 2020.

Year Interval	APC (95% CI)
Overall	
1999-2005	41.6738 (30.5419-53.7549)
2005-2012	4.3602 (0.2462-8.643)
2012-2020	-1.2319 (-3.3939- 0.9785)
Men	
1999-2005	39.5556 (29.5632-50.3188)
2005-2012	4.0585(0.2-8.0657)
2012-2020	-1.397(-3.4503-0.7001)
Women	
1999-2005	42.5805(32.1097-53.8812)
2005-2012	0.7648(8.4895-2.5873)
2012-2020	-3.223(0.9762--1.1637)
NH White	
1999-2005	42.2846(31.8082-53.5936)
2005-2012	4.9247(1.0381-8.9609)
2012-2020	-0.8857(-3.0039-1.2787)
NH Black or African American	
1999-2005	34.3614(23.4472-46.2405)
2005-2012	4.3481(-0.2628-9.1722)
2012-2020	-1.0828(-3.5108-1.4064)
NH American Indian or Alaskan native	
1999-2005	36.0586(23.2038-50.2546)
2005-2020	0.2466(-0.9948-1.5035)
Hispanic or Latino	
1999-2005	34.3935(23.1384-46.6775)
2005-2020	-0.4679(-1.4956-0.5705)
NH Asian or Pacific Islander	
1999-2007	32.7032(25.7815-40.0058)
2007-2020	-1.1477(-2.1288--0.1568)
Non-Metropolitan Areas	
1999-2004	42.6354(30.3-56.1386)
2004-2012	8.3534(5.5481-11.2334)
2012-2020	-0.212(-1.9498-1.5565)
Metropolitan areas	
1999-2005	41.7507(31.1482-53.2103)
2005-2011	4.3556(-0.8195-9.8006)
2011-2020	-1.1818(-2.9451-0.6135)

**Table 3:** Overall and sex‐stratified cancer and psychoactive substance use- related age-adjusted mortality rates (AAMR) per 100,000 in the United States, 1999 to 2020.

Year	Males	Females	Overall
1999	8.23 (8.03–8.44)	3.66 (3.55–3.78)	5.66 (5.54–5.77)
2000	11.33 (11.09–11.57)	4.99 (4.86–5.13)	7.72 (7.59–7.84)
2001	10.65 (10.42–10.88)	4.91 (4.77–5.04)	7.36 (7.24–7.49)
2002	10.55 (10.32–10.77)	4.96 (4.83–5.10)	7.35 (7.23–7.47)
2003	28.24 (27.87–28.61)	13.51 (13.29–13.73)	19.79 (19.59–19.99)
2004	38.78 (38.36–39.21)	18.86 (18.60–19.12)	27.31 (27.07–27.54)
2005	49.19 (48.71–49.67)	23.79 (23.50–24.08)	34.58 (34.32–34.84)
2006	51.94 (51.45–52.42)	25.66 (25.36–25.96)	36.89 (36.62–37.15)
2007	55.28 (54.78–55.78)	27.92 (27.61–28.23)	39.65 (39.37–39.92)
2008	60.13 (59.62–60.65)	30.45 (30.13–30.77)	43.19 (42.9–43.47)
2009	58.37 (57.87–58.87)	29.69 (29.37–30.00)	42.05 (41.77–42.33)
2010	63.23 (62.72–63.75)	31.91 (31.58–32.23)	45.43 (45.14–45.71)
2011	63.56 (63.06–64.07)	32.48 (32.16–32.81)	46 (45.71–46.28)
2012	66.51 (66–67.03)	33.92 (33.59–34.24)	48.12 (47.83–48.41)
2013	65 (64.5–65.5)	33.26 (32.94–33.58)	47.14 (46.85–47.42)
2014	64.6 (64.11–65.09)	33.02 (32.71–33.34)	46.89 (46.62–47.17)
2015	64.82 (64.34–65.31)	33.61 (33.29–33.92)	47.32 (47.05–47.6)
2016	64.16 (63.68–64.64)	33.28 (32.97–33.59)	46.92 (46.65–47.19)
2017	62.92 (62.45–63.39)	32.70 (32.40–33.00)	46.04 (45.77–46.3)
2018	61.9 (61.44–62.36)	32.30 (32.00–32.60)	45.37 (45.11–45.63)
2019	60.83 (60.38–61.27)	31.61 (31.31–31.90)	44.58 (44.33–44.84)
2020	59 (58.57–59.44)	30.72 (30.44–31.01)	43.26 (43.01–43.51)

### 3.2 Gender stratified trends in AAMR for psychoactive substance use-related mental disorders and cancer

Throughout the study period, males consistently exhibited higher AAMRs than females (overall AAMR: males 51.57; females 25.80). For males, AAMR rose from 1999 to 2005 (APC: 39.56, 95% CI: 29.56–50.32), continued increasing in 2012 (APC: 4.06, 95% CI: 0.2–8.07), and then showed stability till 2020 (APC: -1.4, 95% CI: -3.45–0.7, p value>0.05). Females followed a similar pattern, with AAMR rising from 1999 to 2005 (APC: 42.58, 95% CI: 32.11–53.88), peaking in 2012 (APC: 4.56, 95% CI: 0.77–8.49), and then declining in 2020 (APC: -1.15, 95% CI: -3.22–0.98) (**Figure 1**; **Tables 2**, **3**).

### 3.3 Race stratified trends in AAMR for psychoactive substance use-related mental disorders and cancer

**Figure 2:**
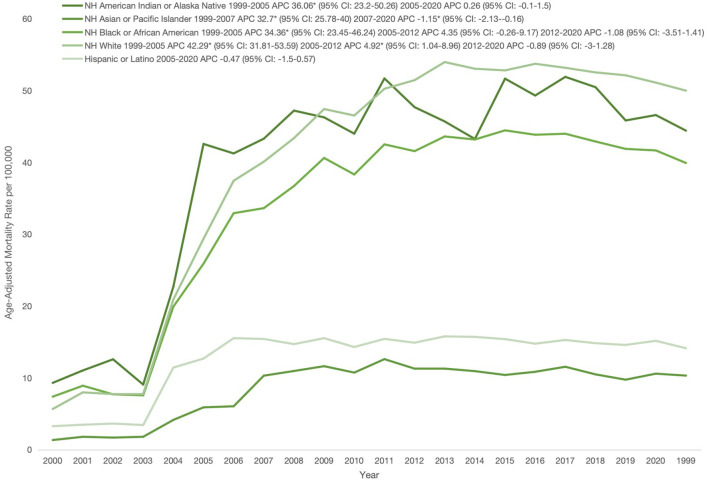
Race stratified age-adjusted mortality rates per 100,000 for psychoactive substance use–related mental disorders and cancer among U.S. adults aged ≥25 years, 1999–2020. *Indicates that the annual percentage change (APC) is significantly different from zero at α, 0.05; APC, annual percent change; CI, confidence interval; NH, non-Hispanic.

When stratified by race, NH American Indian or Alaska Native individuals had the highest AAMR (41.45), followed by NH White (40.78), NH Black or African American (35.17), Hispanic or Latino (13.63), and NH Asian or Pacific Islander populations, who had the lowest (9.43). From 1999 to 2005, NH American Indian or Alaska Native populations experienced a marked increase (APC: 36.06; 95% CI: 23.20–50.25), followed by stability through 2020 (APC: 0.25; 95% CI: -0.99–1.50). Hispanic populations showed a similar pattern, with an initial rise from 1999 to 2005 (APC: 34.39; 95% CI: 23.14–46.68) and stability thereafter (APC: -0.47; 95% CI: -1.50–0.57). Among NH Asian or Pacific Islanders, AAMR rose sharply from 1999 to 2007 (APC: 32.7; 95% CI: 25.78–40.01), then declined until 2020 (APC: -1.15; 95% CI: -2.13 to -0.16). NH White and NH Black populations both demonstrated increases from 1999 to 2005 (NH White: APC 42.28, 95% CI: 31.81–53.59; NH Black: APC 34.36, 95% CI: 23.45–46.24). Thereafter, NH White AAMR continued rising until 2012 (APC: 4.92; 95% CI: 1.04–8.96), followed by stability through 2020 (APC: -0.89; 95% CI: -3.00–1.29), while NH Black AAMR plateaued between 2005 and 2012 (APC: 4.35; 95% CI: -0.26–9.17) and remained stable through 2020 (APC: -1.08; 95% CI: -3.51–1.40). (**Figure 2**; **Tables 2**, **4**).

**Table 4:** Cancer and psychoactive substance use– related age-adjusted mortality rates (AAMR) per 100,000, stratified by race in the United States, 1999 to 2020.

Year	Black or African American	NH American Indian or Alaskan Native	Hispanic or Latino	NH Asian or Pacific Islander	NH White
1999	7.4 (6.97–7.82)	9.32 (7.26–11.77)	3.3 (2.9–3.7)	1.36 (1.02–1.78)	5.68 (5.55–5.80)
2000	8.92 (8.46–9.39)	11.05 (8.84–13.65)	3.5 (3.1–3.9)	1.81 (1.44–2.26)	8 (7.85–8.14)
2001	7.72 (7.29–8.15)	12.6 (10.24–14.96)	3.7 (3.3–4)	1.7 (1.35–2.12)	7.75 (7.61–7.89)
2002	7.58 (7.16–7.99)	9.08 (7.17–11.35)	3.4 (3.1–3.8)	1.81 (1.46–2.22)	7.78 (7.64–7.93)
2003	19.93 (19.26–20.61)	22.71 (19.45–25.96)	11.4 (10.8–12.1)	4.16 (3.61–4.7)	20.93 (20.7–21.16)
2004	25.91 (25.14–26.67)	42.58 (38.25–46.9)	12.7 (12–13.4)	5.91 (5.27–6.54)	29.38 (29.11–29.65)
2005	32.94 (32.09–33.79)	41.26 (37.09–45.43)	15.5 (14.8–16.2)	6.06 (5.44–6.68)	37.46 (37.16–37.76)
2006	33.64 (32.8–34.49)	43.31 (39.02–47.61)	15.4 (14.7–16.1)	10.32 (9.54–11.11)	40.11 (39.8–40.42)
2007	36.72 (35.84–37.6)	47.22 (42.8–51.64)	14.7 (14.1–15.4)	10.97 (10.17–11.76)	43.39 (43.07–43.72)
2008	40.63 (39.72–41.55)	46.28 (42.01–50.55)	15.5 (14.9–16.2)	11.64 (10.84–12.45)	47.44 (47.11–47.78)
2009	38.33 (37.45–39.21)	44.01 (40–48.02)	14.3 (13.7–14.9)	10.75 (9.99–11.5)	46.54 (46.21–46.87)
2010	42.52 (41.6–43.43)	51.7 (47.34–56.06)	15.4 (14.8–16.1)	12.62 (11.82–13.42)	50.26 (49.92–50.60)
2011	41.58 (40.69–42.47)	47.7 (43.67–51.73)	14.9 (14.3–15.5)	11.3 (10.57–12.02)	51.46 (51.12–51.80)
2012	43.62 (42.72–44.52)	45.71 (41.89–49.52)	15.8 (15.2–16.4)	11.3 (10.59–12)	53.98 (53.64–54.33)
2013	43.2 (42.32–44.07)	43.27 (39.6–46.93)	15.7 (15.1–16.3)	10.95 (10.28–11.62)	53.05 (52.7–53.39)
2014	44.47 (43.6–45.35)	51.67 (47.75–55.59)	15.4 (14.8–16)	10.42 (9.79–11.06)	52.82 (52.48–53.15)
2015	43.86 (43.01–44.72)	49.32 (45.6–53.04)	14.8 (14.2–15.3)	10.86 (10.23–11.48)	53.74 (53.4–54.08)
2016	44 (43.15–44.84)	51.92 (48.24–55.6)	15.3 (14.8–15.8)	11.56 (10.92–12.19)	53.18 (52.85–53.52)
2017	42.92 (42.1–43.74)	50.48 (46.89–54.06)	14.8 (14.3–15.4)	10.5 (9.92–11.08)	52.54 (52.21–52.86)
2018	41.89 (41.09–42.69)	45.85 (42.53–49.17)	14.6 (14.1–15.1)	9.76 (9.21–10.31)	52.14 (51.82–52.47)
2019	41.68 (40.9–42.46)	46.6 (43.36–49.85)	15.2 (14.7–15.7)	10.6 (10.05–11.16)	51.12 (50.8–51.44)
2020	39.93 (39.17–40.68)	44.43 (41.31–47.54)	14.1 (13.7–14.6)	10.33 (9.79–10.86)	50 (49.69–50.31)

### 3.4 Geographical regions stratified trends in AAMR for psychoactive substance use-related mental disorders and cancer

Substantial geographic variation in AAMRs was observed across states and regions. Vermont recorded the highest rate (78.94; 95% CI: 77.26–80.62), while California had the lowest (5.23; 95% CI: 5.16–5.29).

**Figure 3:**
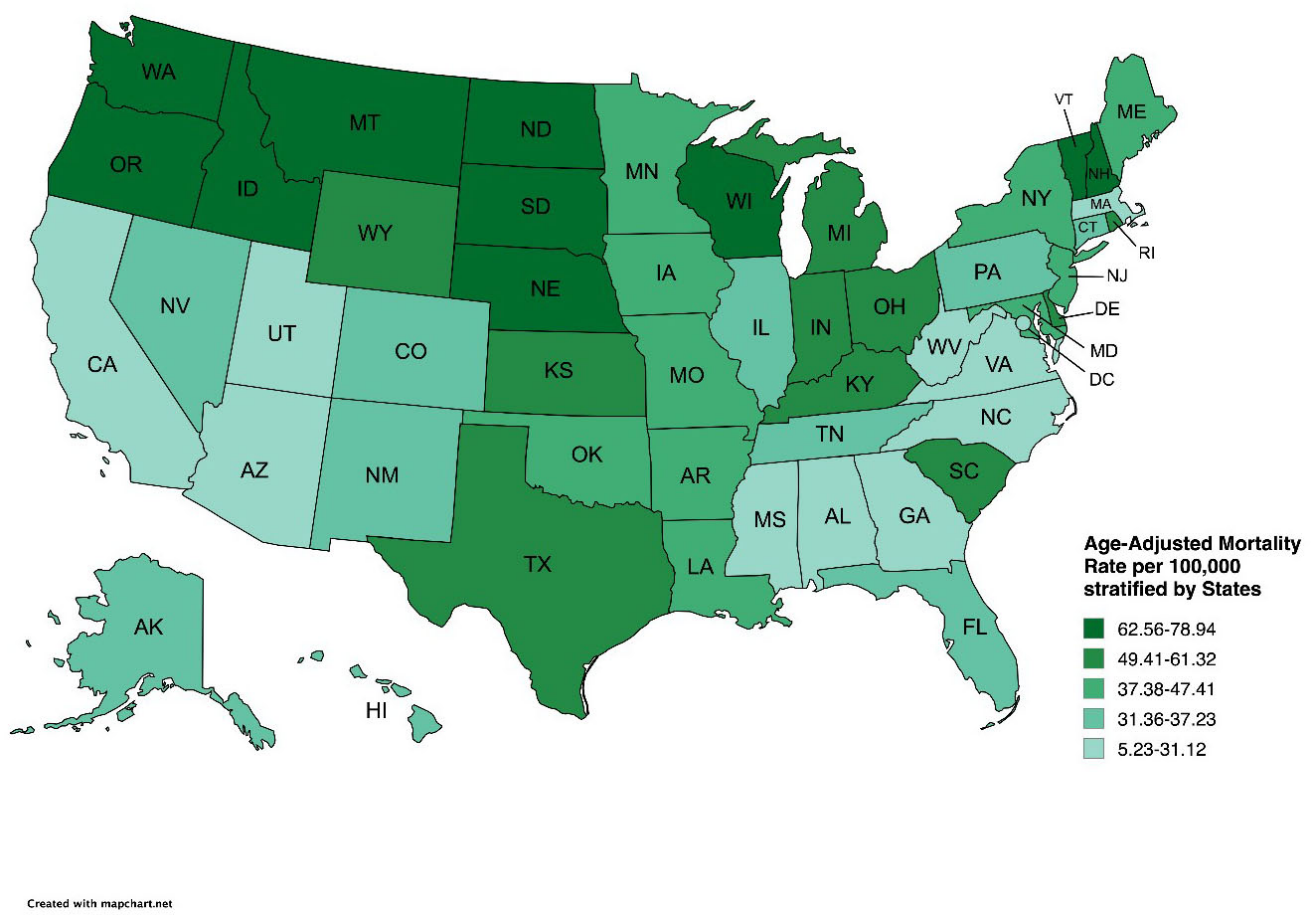
States stratified age-adjusted mortality rates per 100,000 for psychoactive substance use–related mental disorders and cancer among U.S. adults aged ≥25 years, 1999–2020.

**Figure 4:**
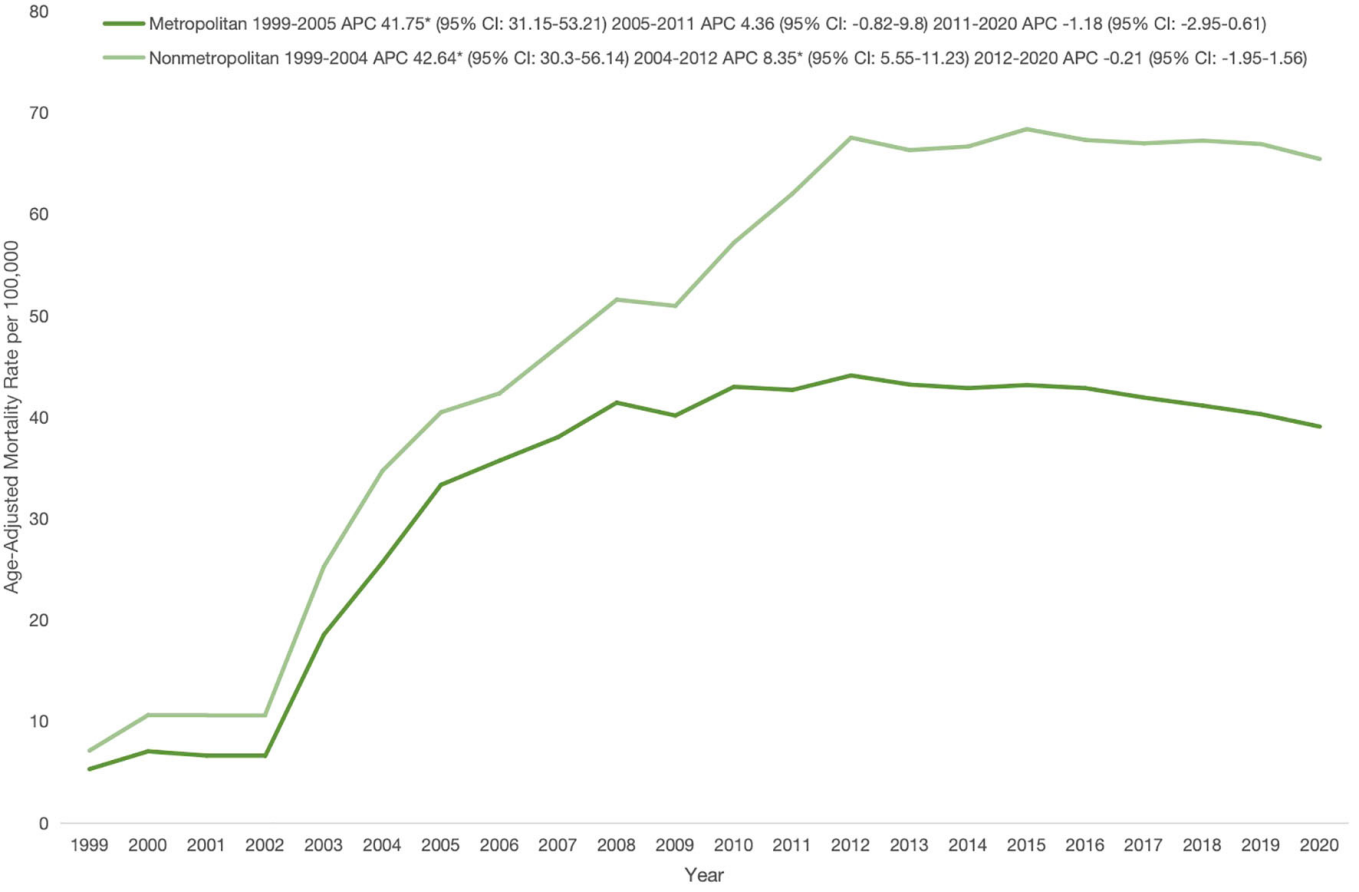
Urbanization stratified age-adjusted mortality rates per 100,000 for psychoactive substance use–related mental disorders and cancer among U.S. adults aged ≥25 years, 1999–2020.* Indicates that the annual percentage change (APC) is significantly different from zero at α, 0.05; APC, annual percent change; CI, confidence interval.

States in the 90th percentile or higher for AAMR included Vermont, Montana, Nebraska, North Dakota, Oregon, and Wisconsin, while those in the 10th percentile or lower included Alabama, Massachusetts, Mississippi, Virginia, and West Virginia. (**Figure 3****;** **Supplementary Table 2****).** Across U.S. regions, the Midwest exhibited the highest AAMR (49.32), followed by the Northeast (37.5), South (35.91), and West (25.38) (**Supplementary Table 3****).** Overall, nonmetropolitan areas had higher AAMRs than metropolitan areas (49.6 *vs*. 34.26). In nonmetropolitan areas, AAMR increased sharply from 1999 to 2004 (APC: 42.64; 95% CI: 30.3–56.14), continued rising through 2012 (APC: 8.35; 95% CI: 5.55–11.23), and then showed stability between 2012 and 2020 (APC: -0.21; 95% CI: -1.95–1.56). Metropolitan areas showed a similar early increase from 1999 to 2005 (APC: 41.75; 95% CI: 31.15–53.2), followed by stability through 2011 (APC: 4.36; 95% CI: -0.82–9.8) continuing until 2020 (APC: -1.18; 95% CI: -2.95–0.61) (**Figure 4**; **Tables 2**, **5****).**

**Table 5:** Cancer and psychoactive substance use related age-adjusted mortality rates (AAMR) per 100,000, stratified by urban-rural classification in the United States, 1999 to 2020.

Year	Metropolitan	Nonmetropolitan
1999	5.31 (5.19–5.43)	7.13 (6.84–7.42)
2000	7.06 (6.93–7.20)	10.63 (10.28–10.98)
2001	6.64 (6.51–6.77)	10.60 (10.25–10.95)
2002	6.61 (6.48–6.74)	10.69 (10.34–11.04)
2003	18.55 (18.34–18.77)	25.26 (24.73–25.80)
2004	25.68 (25.43–25.93)	34.70 (34.08–35.32)
2005	33.32 (33.04–33.61)	40.47 (39.81–41.14)
2006	35.71 (35.42–36.00)	42.34 (41.66–43.01)
2007	38.03 (37.73–38.33)	46.94 (46.23–47.64)
2008	41.42 (41.11–41.73)	51.57 (50.84–52.31)
2009	40.15 (39.85–40.45)	50.95 (50.22–51.67)
2010	42.98 (42.67–43.29)	57.18 (56.42–57.95)
2011	42.67 (42.37–42.97)	62.01 (61.22–62.80)
2012	44.10 (43.79–44.40)	67.53 (66.71–68.35)
2013	43.20 (42.90–43.50)	66.30 (65.49–67.10)
2014	42.85 (42.56–43.14)	66.66 (65.86–67.46)
2015	43.15 (42.86–43.44)	68.37 (67.56–69.18)
2016	42.85 (42.57–43.14)	67.30 (66.50–68.09)
2017	41.92 (41.64–42.20)	66.97 (66.18–67.76)
2018	41.13 (40.86–41.40)	67.24 (66.46–68.03)
2019	40.28 (40.01–40.54)	66.90 (66.12–67.67)
2020	39.06 (38.81–39.32)	65.43 (64.67–66.20)

### 3.5 Subgroup analysis for psychoactive substance use-related mental disorders and cancer stratified by substance type

**Figure 5:**
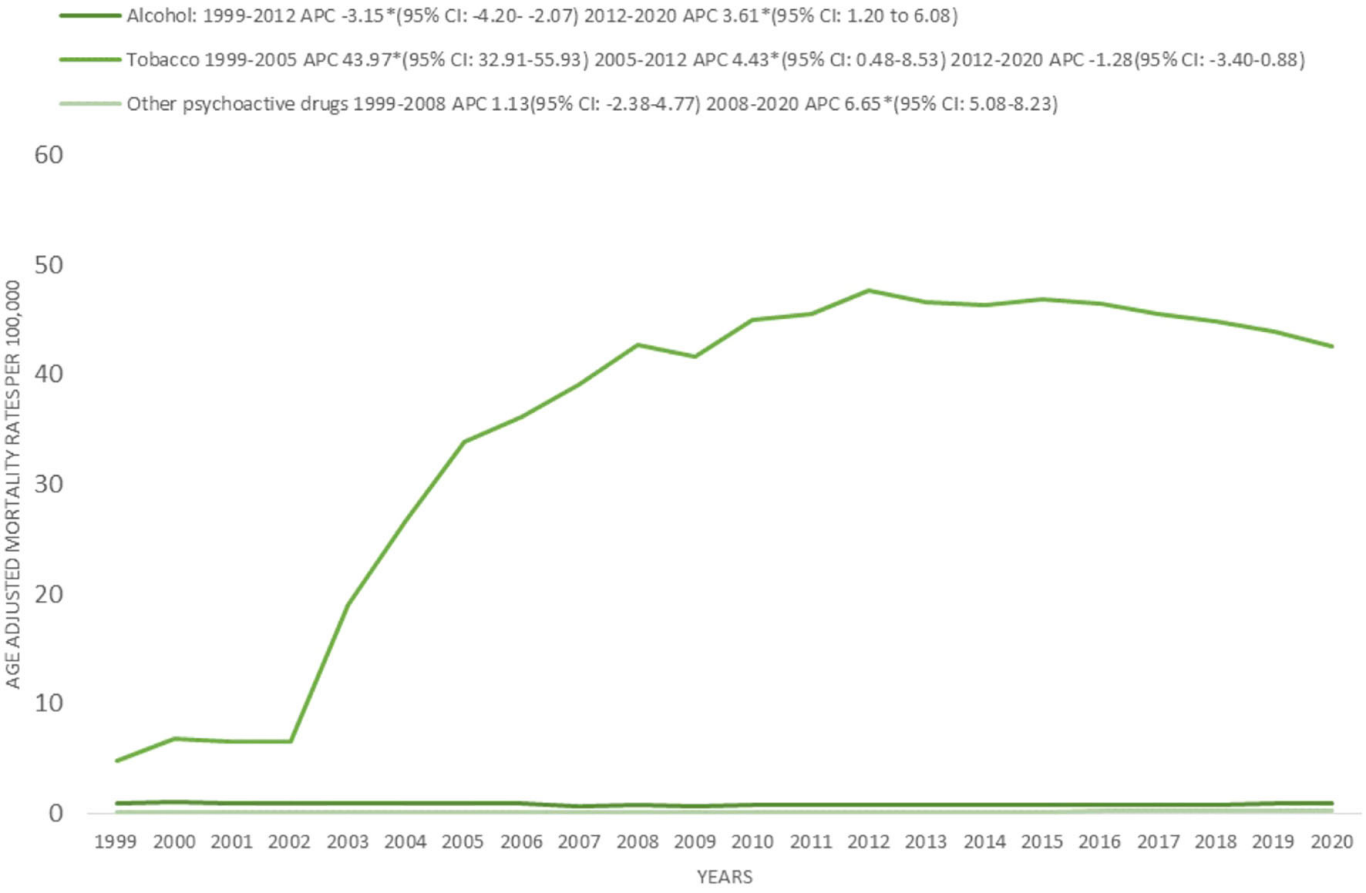
Psychoactive drug type stratified, age-adjusted mortality rates per 100,000 for psychoactive substance use–related mental disorders and cancer among U.S. adults aged ≥25 years, 1999–2020. *Indicates that the annual percentage change (APC) is significantly different from zero at α, 0.05; APC, annual percent change; CI, confidence interval.

The highest AAMRs were associated with tobacco, alcohol, opioids, and multiple drug use (including other psychoactive substances). The total number of deaths are as follows (Tobacco= 1,760,843; Alcohol= 40,793; Other psychoactive drugs= 6776; Opioids= 1395). From 1999–2012, the AAMR related to concomitant alcohol and cancer declined significantly (APC: –3.15; 95% CI: –4.20 to –2.07). However, between 2012 and 2020, this trend reversed, with a significant increase observed (APC: 3.61; 95% CI: 1.20-6.08). For tobacco use, AAMR rose sharply from 1999–2005 (APC: 43.97; 95% CI: 32.91 to 55.93), followed by a slower but continued increase until 2012 (APC: 4.43; 95% CI: 0.48 to 8.53), and subsequently stabilized from 2012–2020 (APC: –1.28; 95% CI: –3.40 to 0.88). In contrast, AAMRs related to other psychoactive drug use remained stable from 1999–2008 (APC: 1.13; 95% CI: –2.38 to 4.77) but then demonstrated a marked increase through 2020 (APC: 6.65; 95% CI: 5.08 to 8.23). The overall AAPCs were as follows: Alcohol: –0.62 (95% CI: -2.59 to 5.94, p > 0.05); Tobacco: 12.03* (95% CI: 9.26 to 14.88, p < 0.05); Other psychoactive drugs: 4.25* (95% CI: -2.59 to 5.94, p < 0.05). (**Figure 5**).

## 4 Discussion

**Figure 6:**
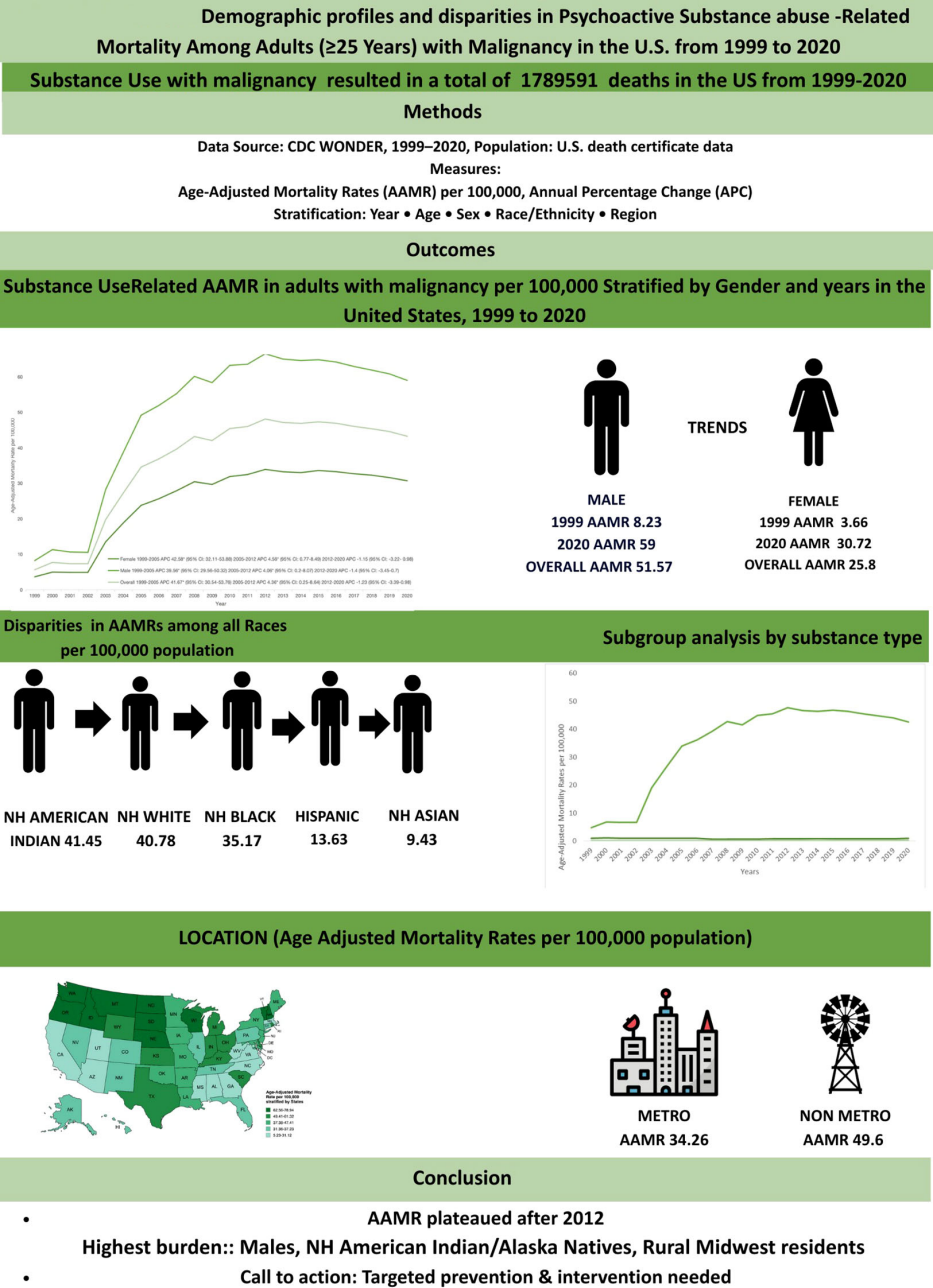
Central illustration: demographic trends and disparities in age-adjusted mortality rates per 100,000 associated with psychoactive substance use-related mental disorders and cancer among US adults aged ≥25, 1999–2020.

In this 22-year nationwide analysis (1999–2020), we observed a substantial rise in AAMRs attributable to psychoactive SU–related mental disorders and cancer. Mortality increased sharply between 1999 and 2005, followed by a slower but sustained rise through 2012, and a period of relative stabilization thereafter. Across all years, males consistently exhibited higher AAMRs than females. Racial and ethnic disparities were pronounced: NH American Indian/Alaska Native individuals experienced the highest mortality burden, whereas Hispanic and Asian/Pacific Islander populations had the lowest rates. Geographic patterns also revealed distinct gradients, with the highest AAMRs observed in the Midwest and nonmetropolitan areas and the lowest in the Western region. Among all psychoactive substances, the greatest mortality burden was associated with tobacco and alcohol-related SU (**Figure 6**).

The early years of the study period were marked by a steep rise in AAMRs, occurring alongside multiple overlapping public health challenges. Increasing tobacco use, heavy alcohol consumption, and expanding illicit drug use; including opioids, cocaine, methamphetamine, and polysubstance exposure, may have collectively contributed to the observed upward trend. National surveys during this period documented rising prevalence of alcohol use disorders (AUDs) and nicotine dependence, both of which are well-established risk factors for multiple cancers (25). Tobacco exposure has historically demonstrated the strongest association with cancer mortality, particularly for lung cancer (26). However, public awareness of alcohol as a cancer risk factor remains limited, with only 38% of U.S. adults recognizing this association (27). In parallel, the opioid crisis evolved in successive waves, beginning with increased prescription opioid use in the late 1990s and followed by rising heroin-related deaths after 2010, patterns described in prior epidemiologic analyses (28). Cancer survivors may represent a particularly vulnerable population, as chronic opioid exposure for pain management and alcohol dependence have been associated with higher mortality in previous studies (17). Additionally, given the long latency of many tobacco-related malignancies, often exceeding a decade, historical smoking patterns from the 1990s and early 2000s may provide epidemiologic context for the rising mortality observed during the early and mid-study period.

Our CDC WONDER analysis showed that cancer and AUD-related AAMRs began rising after 2012, possibly reflecting an increasing disease burden, although changes in coding and reporting practices cannot be excluded. National data similarly report an 83% increase in AUD diagnoses among cancer survivors between 2012 and 2021 (16), a trend described in prior studies in the context of evolving alcohol availability, social norms, and pandemic-related stressors (29–32). Because alcohol is a Group 1 carcinogen associated with liver, colorectal, oral, pharyngeal, and female breast cancers, the rising prevalence of AUD may provide epidemiologic context for the mortality patterns observed in this population. Surveys indicate that up to one-third of cancer survivors exceed recommended alcohol limits and nearly one-fifth meet criteria for misuse (15, 33), suggesting that the growing AUD burden among survivors could be clinically relevant, although causal inferences cannot be established from the present analysis.

A major driver of the mortality trends observed in this study is the changing landscape of tobacco-related malignancies. While lung cancer incidence has declined by 3% annually in men since 2012 (26), mortality remains the most reliable measure of progress because it is less susceptible to lead-time bias and over-diagnosis compared to incidence data (34–36). Reductions in smoking, likely influenced by strengthened tobacco control policies and public education campaigns (37), alongside advances in treatment and broader screening uptake, have coincided with a 34% decline in overall cancer mortality from 1991 to 2022 (26). Recent data indicate that mortality declines have accelerated for both smoking-related and smoking-unrelated lung cancers, though progress in the latter remains slower, possibly due to weaker incidence effects (11, 38). Updated American Cancer Society (ACS) guidelines expanding lung cancer screening eligibility to an additional five million former smokers may further influence these trends (39). Simultaneously, stricter opioid-prescribing regulations and major legal settlements, as well as harm-reduction efforts including widespread naloxone distribution, have been associated with reductions in iatrogenic misuse and opioid-related deaths (40). Despite these advances, lung cancer remains the leading cause of cancer-related mortality, exceeding colorectal, breast, and prostate cancers combined in 2022 (26). While smoking remains the leading preventable cause of death in the United States, with approximately 85% of lung cancer deaths associated with cigarette smoking (41), shifts in population demographics specifically an increasing proportion of older adults and former smokers, correlate substantially with overall mortality trends (42). Additionally, the increasing prevalence of e-cigarette and heated tobacco product use among younger adults has prompted concern regarding future health outcomes. These products are associated with biomarkers of oxidative stress and DNA damage (43), which are frequently identified in the context of carcinogenic processes.

Elevated AAMRs among males (51.57 versus 25.80) are consistent with evidence identifying male sex as a persistent marker for cancer and SU-related mortality. These trends align with observed environmental and lifestyle exposures, including higher documented frequencies of tobacco and alcohol use, as well as differential biological profiles involving endogenous hormone exposure and immune response (44, 45). Despite this baseline difference, females exhibited a similar temporal pattern of rapid increases from 1999 to 2012. This parallel rise may be contextualized by surveillance data indicating a pronounced increase in overdose mortality among females, alongside rising trends in binge and heavy alcohol consumption among those younger than 50 years (46). Additionally, these rising mortality patterns are observed alongside broader systemic factors, including increasing psychiatric comorbidities, shifting metabolic risk profiles, psychosocial barriers to accessing early SU treatment and routine oncological screening (47–49). Furthermore, the recent stabilization in female AAMRs occurs in tandem with complex, ongoing shifts in late-stage cancer outcomes; while historical declines in breast cancer mortality have slowed (26), mortality from pancreatic and lung cancers is increasing. Together, these intersecting trends suggest a clear need for sex-specific public health strategies to address SU-related cancer mortality (45, 50).

The highest AAMRs for SU- and cancer-related mortality were observed among NH American Indians/Alaska Natives. This trend may reflect rising tobacco-related cancer incidence (51), potentially compounded by culturally rooted tobacco use and lower cessation rates relative to other racial and ethnic groups (52, 53). These racial disparities are often intertwined with geographic inequities; for instance, a shortage of clinicians trained to manage co-occurring psychiatric conditions and tobacco dependence is particularly acute in non-metropolitan regions. Furthermore, the increasing prevalence of SU in rural populations where alcohol and illicit drug use now rival or exceed urban levels is frequently associated with geographic isolation, stigma, limited anonymity, and restricted access to treatment (54, 55). Addressing these inequities necessitates culturally tailored prevention and cessation programs, alongside expanded access to screening and early detection services (56). Conversely, Hispanic and NH Asian American populations exhibited the lowest overall AAMRs in our analysis. The attenuated mortality burden observed among NH Asian Americans aligns with prior literature documenting lower reported alcohol consumption within these cohorts (57). Similarly, the correspondingly low rates among Hispanic populations are consistent with the ‘Hispanic paradox.’ This phenomenon is frequently contextualized by healthier immigrant selection and lower smoking prevalence among foreign-born individuals, although the potential under-ascertainment of mortality data within this group remains an important methodological consideration (58).

Building on these observations, future research should prioritize large-scale epidemiologic studies to further clarify the association between SU and cancer outcomes. While our study utilizes cross-sectional data, prospective cohort studies are needed to evaluate the longitudinal relationships between tobacco, alcohol, opioids, and other psychoactive substances with cancer progression and treatment response. To enhance the precision of trend monitoring, expanding population-level surveillance by integrating cancer registries with SU and mental health data is essential. Such linked datasets would allow for real-time identification of emerging high-risk populations and shifting geographic disparities. Special attention should be directed toward the vulnerable groups identified in this analysis, including NH American Indian/Alaska Native individuals, and residents of non-metropolitan areas. Understanding the specific structural barriers faced by these groups is critical for guiding targeted prevention efforts.

Finally, evaluating the impact of public health policies including tobacco control, medication-assisted therapy for opioid use disorder, and harm-reduction programs on cancer-specific mortality may inform more effective, evidence-based interventions. A multidisciplinary approach that bridges oncology, public health, and addiction medicine will be vital to addressing the dual burden of malignancy and SU-related mortality.

### 4.1 Limitations

This study has several limitations. It relies on death certificate data from the CDC WONDER database, which is subject to inherent limitations, including potential bias and misclassification in defining of both cancers and SU-related disorders as causes of death. Variability in physician reporting, diagnostic coding practices, and the ICD-9 to ICD-10 transition may have contributed to classification bias. This study is limited by the lack of individual-level data (labs, comorbidities, treatments, social determinants), potential effects of migration on state-level mortality, possible masking of intra-county differences by metropolitan classifications. The ecological design precludes causal inference at the individual level, as observed associations may be confounded by unmeasured variables. Geographic differences should be interpreted cautiously, as variations may reflect differences in demographics, reporting accuracy, healthcare access, or SU service availability rather than true risk. Subgroup analysis for opioids was not feasible due to unstable and unreliable estimates across multiple years. Despite these limitations, our study offers valuable national insights into long-term trends in cancer and SU–related mortality, highlighting demographic and geographic disparities and temporal patterns over two decades.

## 5 Conclusion

Our 22-year analysis of national mortality data reveals a significant evolution in the landscape of concomitant psychoactive SU-related mental disorders and cancer mortality in the United States. The overall AAMR increased sharply from 1999 to 2005, continued to rise gradually until 2012, and then plateaued through 2020, indicating stabilization in recent years. Despite this overall trend, our findings underscore persistent and significant demographic and geographic disparities, with a disproportionately higher burden observed among males, NH American Indian/Alaska Native individuals, and residents of non-metropolitan regions in the Midwest. Furthermore, the divergent patterns identified across specific substance categories, notably the late-period rise in alcohol-related mortality and the shifting trends in tobacco-related deaths point to the multifaceted nature of substance-associated cancer mortality. Our results highlight the importance of considering specialized outreach and integrated screening strategies tailored to high-burden demographic groups and rural communities. Future research should focus on exploring the underlying social and structural factors associated with these observed disparities to better inform targeted public health interventions and health equity initiatives within the cancer care continuum.

## Data Availability

The original contributions presented in the study are included in the article/**Supplementary Material**, further inquiries can be directed to the corresponding author/s.
