# Supplementary material for: Regional, demographic, and temporal trends in psychoactive substance use-related mental disorder and cancer mortality in U.S. adults: a nationwide CDC WONDER analysis (1999–2020)
Source: Front Oncol. 2026 Mar 18;16:1753582. doi: 10.3389/fonc.2026.1753582 (PMC13038565; doi:10.3389/fonc.2026.1753582)
Supplement: Supplementary file 1 [file Table1.docx]

**Supplementary Material**

**Supplemental Table 1: Cancer and psychoactive substance use related deaths stratified by place of death in the United States, 1999 to 2020**

| **Year** | **Medical Facility** | **Nursing Home or Long-term Care Facility** | **Hospices** | **Home** | **Place of death unkown** | **Other** |
| --- | --- | --- | --- | --- | --- | --- |
| **1999** | 4362 | 1465 | missing | 3744 | missing | 423 |
| **2000** | 5819 | 1970 | missing | 5469 | missing | 586 |
| **2001** | 5529 | 1927 | missing | 5337 | missing | 663 |
| **2002** | 5667 | 1897 | missing | 5385 | missing | 742 |
| **2003** | 14923 | 4690 | 302 | 14600 | 60 | 2824 |
| **2004** | 20533 | 6851 | 682 | 20927 | 103 | 3436 |
| **2005** | 25354 | 8518 | 3118 | 26684 | 104 | 3967 |
| **2006** | 27011 | 9388 | 4362 | 29102 | 292 | 3442 |
| **2007** | 28459 | 10348 | 5923 | 31892 | 149 | 3940 |
| **2008** | 31158 | 11344 | 7491 | 35163 | 206 | 4379 |
| **2009** | 29741 | 10952 | 8098 | 35658 | 435 | 4366 |
| **2010** | 31857 | 11932 | 9534 | 39765 | 28 | 4937 |
| **2011** | 32237 | 12543 | 10445 | 41815 | 28 | 4975 |
| **2012** | 33767 | 12835 | 12138 | 45517 | 41 | 5446 |
| **2013** | 32703 | 13245 | 12583 | 46138 | 51 | 5581 |
| **2014** | 33018 | 12761 | 14087 | 48171 | 71 | 4538 |
| **2015** | 33340 | 13168 | 15919 | 50502 | 47 | 3718 |
| **2016** | 33921 | 13106 | 16247 | 51109 | 26 | 3852 |
| **2017** | 33780 | 13302 | 16201 | 51928 | 22 | 3907 |
| **2018** | 34143 | 13011 | 16765 | 52287 | 25 | 4023 |
| **2019** | 34020 | 13267 | 16501 | 52769 | 29 | 4245 |
| **2020** | 30880 | 10120 | 13471 | 60686 | 25 | 4670 |
| **total** | 562,922 | 208640 | 183867 | 754648 | 1754 | 78660 |

**Supplemental Table 2: Cancer and psychoactive substance use– related age-adjusted mortality rates (AAMR) per 100,000, stratified by states in the United States, 1999 to 2020**

| **States** | **AAMR( 95%CI)** |
| --- | --- |
| **Alabama** | 12.07 (11.82–12.31) |
| **Alaska** | 36.58 (35.1–38.06) |
| **Arizona** | 26.75 (26.44–27.07) |
| **Arkansas** | 37.38 (36.83–37.92) |
| **California** | 5.23 (5.16–5.29) |
| **Colorado** | 31.43 (31.01–31.86) |
| **Connecticut** | 37.23 (36.75–37.72) |
| **Delaware** | 49.41 (48.29–50.54) |
| **District of Columbia** | 27.1 (25.99–28.21) |
| **Florida** | 33.85 (33.66–34.04) |
| **Georgia** | 26.11 (25.83–26.39) |
| **Hawaii** | 33.58 (32.83–34.34) |
| **Idaho** | 64 (62.96–65.04) |
| **Illinois** | 31.36 (31.11–31.61) |
| **Indiana** | 50.01 (49.57–50.45) |
| **Iowa** | 42.63 (42.07–43.19) |
| **Kansas** | 51.42 (50.75–52.09) |
| **Kentucky** | 51.15 (50.61–51.68) |
| **Louisiana** | 41.92 (41.43–42.4) |
| **Maine** | 43.83 (43–44.65) |
| **Maryland** | 47.41 (46.95–47.88) |
| **Massachusetts** | 14.99 (14.76–15.22) |
| **Michigan** | 59.5 (59.13–59.88) |
| **Minnesota** | 37.81 (37.39–38.23) |
| **Mississippi** | 12.2 (11.88–12.52) |
| **Missouri** | 43.49 (43.07–43.9) |
| **Montana** | 68.13 (66.89–69.37) |
| **Nebraska** | 66.71 (65.77–67.66) |
| **Nevada** | 33.8 (33.21–34.39) |
| **New Hampshire** | 63.42 (62.36–64.49) |
| **New Jersey** | 43.41 (43.06–43.75) |
| **New Mexico** | 35.94 (35.27–36.6) |
| **New York** | 38.56 (38.34–38.77) |
| **North Carolina** | 31.12 (30.84–31.41) |
| **North Dakota** | 78.1 (76.46–79.73) |
| **Ohio** | 54.32 (53.99–54.65) |
| **Oklahoma** | 47.35 (46.79–47.9) |
| **Oregon** | 75.47 (74.79–76.15) |
| **Pennsylvania** | 37.02 (36.77–37.27) |
| **Rhode Island** | 51.5 (50.46–52.55) |
| **South Carolina** | 51.75 (51.24–52.27) |
| **South Dakota** | 64.07 (62.72–65.41) |
| **Tennessee** | 35.02 (34.66–35.39) |
| **Texas** | 49.46 (49.21–49.7) |
| **Utah** | 29.01 (28.41–29.62) |
| **Vermont** | 78.94 (77.26–80.62) |
| **Virginia** | 15.41 (15.18–15.63) |
| **Washington** | 62.56 (62.07–63.05) |
| **West Virginia** | 19.61 (19.14–20.09) |
| **Wisconsin** | 65.71 (65.19–66.23) |
| **Wyoming** | 61.32 (59.64–63.01) |

**Supplemental Table 3: Cancer and psychoactive substance use– related age-adjusted mortality rates (AAMR) per 100,000, stratified by census region in the United States, 1999 to 2020**

| **Census Region** | **Year** | **Age Adjusted Rate (95% CI)** |
| --- | --- | --- |
| **Northeast** | 1999 | 3.25 (3.07–3.44) |
| **Northeast** | 2000 | 4.92 (4.70–5.15) |
| **Northeast** | 2001 | 4.93 (4.70–5.15) |
| **Northeast** | 2002 | 4.87 (4.65–5.09) |
| **Northeast** | 2003 | 18.7 (18.27–19.13) |
| **Northeast** | 2004 | 32.04 (31.47–32.60) |
| **Northeast** | 2005 | 35.86 (35.26–36.45) |
| **Northeast** | 2006 | 49.52 (48.82–50.21) |
| **Northeast** | 2007 | 50.79 (50.08–51.49) |
| **Northeast** | 2008 | 49.67 (48.98–50.36) |
| **Northeast** | 2009 | 48.2 (47.52–48.88) |
| **Northeast** | 2010 | 47.41 (46.75–48.08) |
| **Northeast** | 2011 | 45.96 (45.31–46.62) |
| **Northeast** | 2012 | 47.89 (47.23–48.55) |
| **Northeast** | 2013 | 47.22 (46.57–47.87) |
| **Northeast** | 2014 | 46.32 (45.68–46.95) |
| **Northeast** | 2015 | 48.62 (47.97–49.27) |
| **Northeast** | 2016 | 46.89 (46.26–47.53) |
| **Northeast** | 2017 | 44.78 (44.17–45.39) |
| **Northeast** | 2018 | 43.71 (43.11–44.31) |
| **Northeast** | 2019 | 43.38 (42.79–43.97) |
| **Northeast** | 2020 | 40.82 (40.25–41.39) |
| **Northeast** | total | 37.5 (37.38–37.63) |
| **Midwest** | 1999 | 5.23 (5.01–5.45) |
| **Midwest** | 2000 | 7.18 (6.92–7.43) |
| **Midwest** | 2001 | 7.02 (6.77–7.27) |
| **Midwest** | 2002 | 7.29 (7.03–7.54) |
| **Midwest** | 2003 | 16.07 (15.69–16.44) |
| **Midwest** | 2004 | 28.34 (27.84–28.83) |
| **Midwest** | 2005 | 33.63 (33.09–34.17) |
| **Midwest** | 2006 | 33.01 (32.47–33.54) |
| **Midwest** | 2007 | 46.15 (45.53–46.78) |
| **Midwest** | 2008 | 60.98 (60.27–61.69) |
| **Midwest** | 2009 | 59.08 (58.38–59.77) |
| **Midwest** | 2010 | 65.35 (64.63–66.08) |
| **Midwest** | 2011 | 72.2 (71.44–72.96) |
| **Midwest** | 2012 | 73.44 (72.68–74.2) |
| **Midwest** | 2013 | 71.28 (70.54–72.02) |
| **Midwest** | 2014 | 70.34 (69.61–71.07) |
| **Midwest** | 2015 | 66.64 (65.94–67.34) |
| **Midwest** | 2016 | 66.65 (65.96–67.35) |
| **Midwest** | 2017 | 65.22 (64.53–65.90) |
| **Midwest** | 2018 | 63.53 (62.86–64.20) |
| **Midwest** | 2019 | 62.00 (61.34–62.65) |
| **Midwest** | 2020 | 61.72 (61.08–62.37) |
| **Midwest** | total | 49.32 (49.18–49.45) |
| **South** | 1999 | 6.24 (6.04–6.43) |
| **South** | 2000 | 9.03 (8.79–9.26) |
| **South** | 2001 | 8.40 (8.18–8.63) |
| **South** | 2002 | 8.07 (7.85–8.28) |
| **South** | 2003 | 24.19 (23.82–24.56) |
| **South** | 2004 | 25.27 (24.90–25.64) |
| **South** | 2005 | 39.23 (38.77–39.70) |
| **South** | 2006 | 37.94 (37.49–38.39) |
| **South** | 2007 | 36.92 (36.49–37.36) |
| **South** | 2008 | 37.61 (37.18–38.05) |
| **South** | 2009 | 36.89 (36.46–37.32) |
| **South** | 2010 | 40.56 (40.11–41.01) |
| **South** | 2011 | 39.77 (39.34–40.21) |
| **South** | 2012 | 43.15 (42.70–43.60) |
| **South** | 2013 | 42.71 (42.27–43.15) |
| **South** | 2014 | 43.71 (43.28–44.15) |
| **South** | 2015 | 45.57 (45.13–46.02) |
| **South** | 2016 | 45.60 (45.17–46.04) |
| **South** | 2017 | 45.69 (45.26–46.12) |
| **South** | 2018 | 46.19 (45.77–46.62) |
| **South** | 2019 | 45.28 (44.86–45.70) |
| **South** | 2020 | 43.75 (43.35–44.16) |
| **South** | total | 35.91 (35.82–36.00) |
| **West** | 1999 | 7.52 (7.24–7.81) |
| **West** | 2000 | 8.97 (8.66–9.28) |
| **West** | 2001 | 8.48 (8.18–8.77) |
| **West** | 2002 | 8.64 (8.35–8.94) |
| **West** | 2003 | 17.40 (16.99–17.82) |
| **West** | 2004 | 25.25 (24.76–25.75) |
| **West** | 2005 | 26.51 (26.01–27.02) |
| **West** | 2006 | 27.56 (27.05–28.07) |
| **West** | 2007 | 27.09 (26.59–27.59) |
| **West** | 2008 | 28.17 (27.67–28.67) |
| **West** | 2009 | 27.53 (27.04–28.02) |
| **West** | 2010 | 31.42 (30.90–31.94) |
| **West** | 2011 | 29.96 (29.46–30.45) |
| **West** | 2012 | 31.14 (30.64–31.64) |
| **West** | 2013 | 30.39 (29.91–30.88) |
| **West** | 2014 | 29.49 (29.02–29.96) |
| **West** | 2015 | 30.44 (29.97–30.91) |
| **West** | 2016 | 30.02 (29.55–30.48) |
| **West** | 2017 | 29.27 (28.82–29.72) |
| **West** | 2018 | 28.08 (27.65–28.51) |
| **West** | 2019 | 27.82 (27.39–28.24) |
| **West** | 2020 | 27.09 (26.67–27.50) |
| **West** | total | 25.38 (25.28–25.47) |
| **Total** |  | 36.92 (36.87–36.98) |
